# Fluorescent Probes and Fluorescence (Microscopy) Techniques — Illuminating Biological and Biomedical Research

**DOI:** 10.3390/molecules171214067

**Published:** 2012-11-28

**Authors:** Gregor P. C. Drummen

**Affiliations:** Bionanoscience and Bio-Imaging Program, Cellular Stress and Ageing Program, BIO&NANO-SOLUTIONS, Helmutstr. 3A, D-40472, Düsseldorf, Germany; Email: gpcdrummen@bionano-solutions.de; Tel.: +49-211-2297-3648; Fax: +49-3222-240-7500

**Keywords:** fluorescence, fluorescence microscopy, fluorochrome, dye, probe, super-resolution, dSTORM, FRET, FRAP, quenching, twisted intramolecular charge transfer, excimer, photoswitching, fluorescent protein, phthalocyanines, pyrene, lithium, quantum dot, fluorenone, flavonoid, pituitary hormone, phagolysosomes, oligothiophene, fusogenic liposomes, hyaluronan

## Abstract

Fluorescence, the absorption and re-emission of photons with longer wavelengths, is one of those amazing phenomena of Nature. Its discovery and utilization had, and still has, a major impact on biological and biomedical research, since it enables researchers not just to visualize normal physiological processes with high temporal and spatial resolution, to detect multiple signals concomitantly, to track single molecules *in vivo*, to replace radioactive assays when possible, but also to shed light on many pathobiological processes underpinning disease states, which would otherwise not be possible. Compounds that exhibit fluorescence are commonly called fluorochromes or fluorophores and one of these fluorescent molecules in particular has significantly enabled life science research to gain new insights in virtually all its sub-disciplines: Green Fluorescent Protein. Because fluorescent proteins are synthesized *in vivo*, integration of fluorescent detection methods into the biological system via genetic techniques now became feasible. Currently fluorescent proteins are available that virtually span the whole electromagnetic spectrum. Concomitantly, fluorescence imaging techniques were developed, and often progress in one field fueled innovation in the other. Impressively, the properties of fluorescence were utilized to develop new assays and imaging modalities, ranging from energy transfer to image molecular interactions to imaging beyond the diffraction limit with super-resolution microscopy. Here, an overview is provided of recent developments in both fluorescence imaging and fluorochrome engineering, which together constitute the “fluorescence toolbox” in life science research.

## 1. Introduction

Luminescence is one of those exquisite and amazing phenomena that Nature has to offer. Luminescence (Latin: *Lumen* = light), whether man-made or created by Nature itself, is the only phenomenon that lightens up “life”, besides the visible radiation from combustion and photon emission as a consequence of nuclear fusion of hydrogen by the yellow dwarf star (G2V) that shines on our planet. Interestingly, a photon produced in the core of the sun will take thousands of years to travel from the core to the surface, but only 8 minutes to reach our planet. Commonly, people identify at least two types of luminescence: fluorescence and phosphorescence. However, luminescent processes comprise a large group of related phenomena that have purely physical, chemical, and/or biological/biochemical origins (Table 1). 

**Table 1:** Overview of various luminescent phenomena.

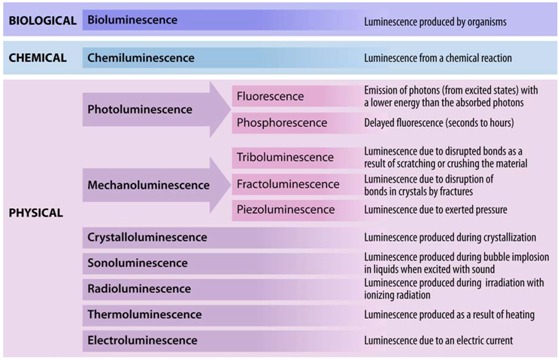

**Figure 1:**
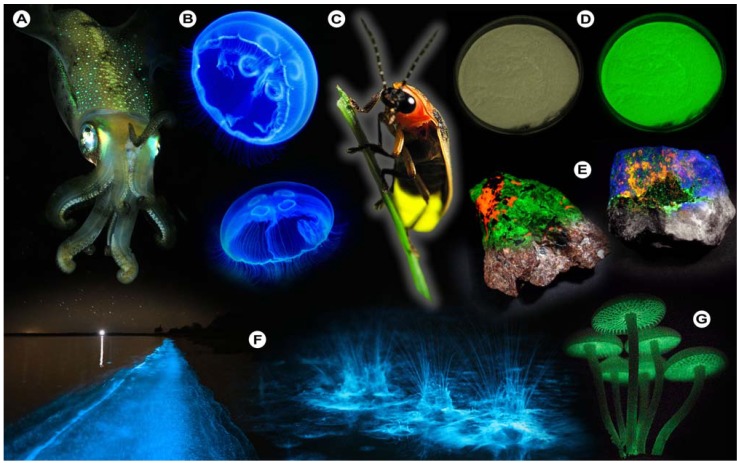
Luminescence in Nature. (**A**) *Sepiolidae* family of squid; (**B**) *Aurelia aurita*, moon jelly fish; please note that the blue glow stems from diffraction and not from bioluminescence; (**C**) *Lampyridae* family of fireflies; (**D**) Phosphorescent zinc sulfide pigment (alkaline earth metal); (**E**) Fluorescent rocks (top is illuminated with UV-light; bottom white light recording): left = Willemite/Calcite; right = Hardystonite (courtesy Ron Teunissen ©2012); (**F**) Luminescent water at the Gippsland Lakes, Australia (courtesy Phil Hart ©2012), which is created by *Noctiluca scintillans*, a non-parasitic marine-dwelling species of dinoflagellate that exhibits bioluminescence; the bioluminescent reaction is instantaneous, as observed in the left picture when the water is mechanically disturbed; (**G**) *Mycena Chlorophos*, a large genus of small saprotrophic mushrooms.

For instance, bioluminescence (Figure 1), the emission of light by organisms, can be found in various cephalopods of the order *Teuthida* (squid), numerous members of the phylum *Cnidaria* (jellyfish), and the *Lampyridae* (fireflies), which all produce light through chemical reactions (chemiluminescence). One of the more astonishing spectacles that may be observed is the nightly glowing of water (Figure 1F), caused by *Noctiluca scintillans* (commonly known as Sea Sparkle), a non-parasitic marine-dwelling dinoflagellate species that is commonly found in shallow waters along the coast and in estuaries. Its bioluminescence stems from a luciferin-luciferase system that is concentrated in spherical organelles (microsources) within the cytosol and is a reaction to mechanical stimulation of *N. scintillans* [1]; similar chemical mechanisms are at work in fireflies and other organisms. 

Generally, deep-sea organisms use their luminescent properties to lure prey, to communicate, to find a mate, or as a defense mechanism, and it is estimated that only a small fraction of the luminescent creatures that dwell in and below the mesopelagic zone (>200–1,000 m depth) of the world’s oceans have been discovered to date. Amongst these bioluminescent entities, however, there is one organism that gained world fame after a fluorescent protein was discovered that has revolutionized life science research: the hydromedusa *Aequorea victoria*, which contains the now famous “Green Fluorescent Protein” (GFP) [2,3,4,5,6,7]. *Aequorea victoria* produces light by the quick release of calcium ions, thereby activating the photoprotein aequorin, which in turn excites GFP. Aequorin is a complex of the 21.4 kDa apoprotein, dioxygen and coelenterazine (luciferin), which results in the oxidative formation of coelenteramide (excited state) and blue emittance at 470 nm when returning to the ground state. Subsequently, GFP absorbs coelenteramide’s blue emission and emits at 505 nm in the green; hence the name Green Fluorescent Protein. The biological function of bioluminescence in jellyfish–bear in mind that continuous overall corporal bioluminescence, as often mistakenly assumed from photographic images (Figure 1B), has not been observed to date–is not well understood, but it is assumed that in some jellyfish species it is used to find a mate or for defense purposes. In other creatures, complex and rapidly changing bioluminescent patterns may be observed, which might constitute a form of communication that currently eludes us. Some squid species have been observed to use bioluminescent flashes to stun potential prey, as recently recorded for the giant squid *Taningia danae* [8].

Luminescent phenomena are not limited to biological systems, but can unexpectedly occur in a myriad of other natural objects and systems. One of these is the capacity of certain solids to emit light due to changes in the crystal structure in response to the exertion of an external force, as in mechanoluminescence (Table 1). Scratching the surface of quartz will induce luminescence to occur where the rock’s surface is disturbed [9,10]. Similarly, the crushing of such ordinary materials such as white crystalline sugar will induce flashes of luminescence [11,12], because bonds are disrupted and the energy is partially dissipated as light. That chemical bonds play important roles in the luminescent properties of certain solids is further exemplified by the fact that photons may equally be produced during crystallization processes [13,14] in which atoms take their place at certain positions in the crystal lattice and bonds are formed. This form of luminescence is aptly called crystalloluminescence.

Ever since the appearance of hominids on this planet and especially *Homo sapiens*, the thinking ape, man has striven to control its environment. The conquest and control of fire did not only change man’s eating habits, but provided light and heat, which allowed *Homo sapiens* to conquer regions in which other hominids could not readily survive, and also gave him an edge in the defense against predators and in the development of a myriad of tools. In modern times, this development continued and the invention of the incandescent light bulb by Thomas Edison in 1879 to date remains one of man’s greatest achievements [15]. 

This invention not only made us independent from the Sun and its illuminating rays, but also had a major impact on scientific research. What would spectroscopy be without a light source, or optical microscopy for that matter of fact? Recent decades have subsequently seen the development of high power and monochromatic light sources, such as lasers and light emitting diodes (LED). The latter will see a bright future, as countries around the world are phasing out the production of conventional incandescent light bulbs in an effort to save energy and reduce global warming (ironically this phasing out also stimulated the use of energy-saving light bulbs that contain harmful and toxic organic compounds and mercury).

As stated previously, the utilization of light-matter interactions has had a major impact on scientific research. It is not only the basis for analytical techniques such as UV/*vis*-, atomic absorption, or infrared spectroscopy, but also for the visualization of microscopic, and more recently even nanoscopic structures via optical microscopy or nanoscopy (super-resolution microscopy). The first scientists who developed optical microscopy for the observation of biology at the microscopic level were Robert Hooke and Antonie van Leeuwenhoek. Hooke developed the compound microscope, which consisted of a stage, a light source and three optical lenses; general features that modern microscopes still contain. With this microscope, Hooke observed insects, such as lice and fleas, plant seeds and plant sections, and published both his biological observations and fundamentals of microscopy in 1665 in the book entitled “Micrographia” [16]. It was Robert Hooke who coined the term “cell” when observing the boxlike porous structure of cork, because it reminded him of the cells of a monastery–from the Latin *cellula*, meaning “a small room”. However, it needs to be noted that Hooke did not observe cells as in our current biological understanding of the word. The observation of single cellular organisms was first achieved by Antonie van Leeuwenhoek in 1678 with a simple microscope containing a single, convex lens that could resolve details as small as 1 μm [17]. His microscope was more difficult to handle than Hooke’s compound version, but with it van Leeuwenhoek observed his “animalcules”: various bacteria, protozoa and spermatozoa, and also the striped patterns in muscle fibers and blood flow in capillaries. These initial pioneering steps led to further development of optical microscopy and consequently major biological discoveries.

The jump from white light microscopy to fluorescence microscopy was by comparison small, and from the beginning of the 20th century, many now prominent names were involved in its development. August Köhler constructed the first ultraviolet (UV) illumination microscope in 1904 at Zeiss Optical Works, but it was Oskar Heimstädt who developed the first rudimentary fluorescence microscope in 1911, with which he studied autofluorescence in organic and inorganic compounds [18]. Improvements were made in 1929 by Philipp Ellinger and August Hirt and their *epi*-fluorescence microscope is still conceptually used in today’s laboratories. With the introduction of lasers (**l**ight **a**mplification through **s**timulated **e**mission of **r**adiation) by Gould, Townes, Schawlow, and Maiman [19,20] in the 1960s, the lack of excitory power was overcome and this paved the way for the development of confocal microscopy. Lasers offered what other light sources could not: a high degree of spatial and temporal coherence, which means that the diffraction limited monochromatic and coherent beam can be focused in a tiny spot, achieving a very high local irradiance. Confocal laser scanning microscopy (CLSM) combines high-resolution optical imaging with depth selectivity [21] and was originally invented by Marvin Minsky in 1957 (reference [22]). Advances in resolution and penetration depth were achieved by multi-photon microscopy, first theoretically described by Maria Göppert-Mayer in 1931 in her doctoral thesis [23], and subsequently further developed by Winfried Denk in the lab of Watt Webb [24].

Concomitant evolution in fluorochrome development allowed fluorescence microscopy to grow beyond the classical boundaries of optical microscopy. Particularly the use and genetic engineering of fluorescent proteins that span the visibly spectrum [7,25,26,27,28], in which the fluorescent properties are controlled, allowed methods such as nanoscopy to flourish, thereby “cheating” Abbe’s diffraction limit [29] and allowing imaging with unsurpassed resolution [30,31]. *In vivo* whole animal imaging was spurred on by developments in nanoparticle technology, especially quantum dots [32,33,34]. Also chemical engineering of classical organic dyes by companies such as Molecular Probes [35] (currently Life Technologies/Invitrogen) meant that for virtually all fields of biological research, probes now became available. This not only led to an increase and evolution in imaging techniques, but also caused new developments in fluorescence spectroscopy, fluorescence multiplexing, high-throughput screening, and the development of simple and fast clinical tests; many of which now can be found in the small labs of local physicians.

This special edition of *Molecules* brings together a number of articles dedicated to fluorescence and its application in life- and biomedical sciences. Collectively, these articles illustrate recent advances in the field and highlight a promising and bright future for life- and biomedical sciences, as well as other, related fields of technology, as concomitant evolution in fluorochrome and imaging technique development rapidly opens up novel avenues of research. In particular, the development of nanoscopy as a “real-time” imaging technique should propel cell biological and biomedical research to new discoveries and a better understanding of both normal and pathological biological processes.

## 2. Fluorescence Techniques and Fluorescence Microscopy

### 2.1. Fluorescence: “Exciting” Luminescence

Luminescence has been known for ages by the term “phosphor”–from *phosphorus*, which means *the light bearer* in ancient Greek–used to designate minerals that glow in the dark after exposure to daylight. Luminescence may be defined as “spontaneous emission of radiation from an electronically or vibrationally excited species not in thermal equilibrium with its environment” [36]. In fact, it makes relatively little difference what type of process causes absorption of a suitable energy quantum–light, radio waves, heat, ionizing radiation, mechanical force, electric current, *etc*. (Table 1)–and subsequent excitation to the excited state. If the material does not dissipate the excess energy via non-radiative processes, such as collision with the surrounding molecules, luminescence will and must occur. 

**Figure 2:**
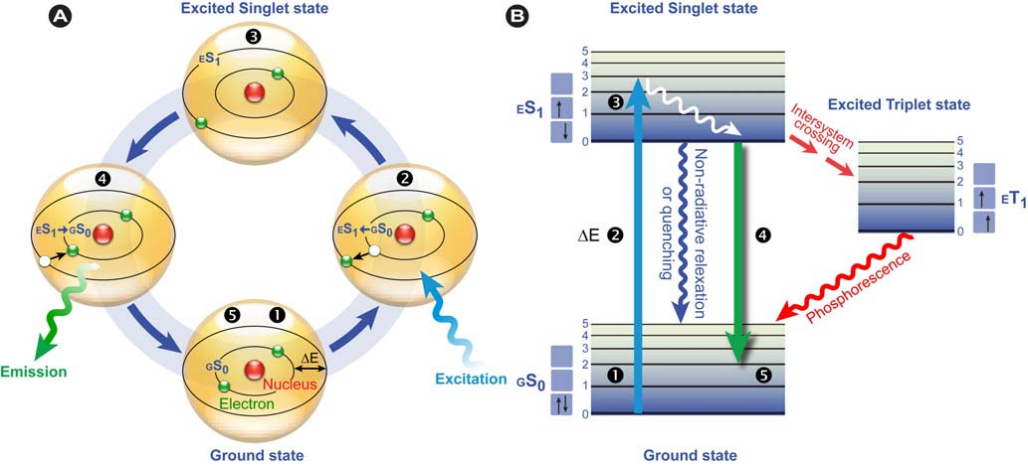
Fluorescence principle. (**A**) Schematic representation of the fluorescence phenomenon in the classical Bohr model. From the ground state *GS*_0_❶, absorption of a light quantum (blue) causes an electron to move to a higher energy level ❷→❸. After residing in this “excited state” ❸ for a particular time, the fluorescence lifetime, the electron falls back to its original level ❸→❹ and the fluorochrome dissipates the excess energy by emitting a photon (green) ❹. (**B**) Jabłoński diagram: Upon photon absorption, a ground state *GS*_0_ electron (electronic singlet) is promoted to a higher and excited state, relaxes quickly to a lower vibrational excited state (white line) and thereby looses energy. When returning to the ground state, it dissipates the remaining energy by emitting a photon with a longer wavelength, *i.e.*, fluorescence emission. The spins of electrons in the singlet states (paired or unpaired anti-parallel spins) compared to the triplet state (unpaired, parallel spin) are depicted. Notice that intersystem crossing from *ES*_1_→ *ET*_1_ requires spin conversion and phosphorescence occurs through relaxation from the triplet excited state.

Of the various luminescent phenomena, photoluminescence in particular has had a major impact on a myriad of scientific and technological disciplines, including chemistry, biology, medicine, physics, and even materials science and nanotechnology [34,37,38]. As stated previously, photoluminescence may be divided into fluorescence and phosphorescence, which both involve the absorption of photons (and their energy), resulting in the promotion of ground state electrons (excitation) to the so-called excited state (Figure 2). This only happens in substances with suitable electron and quantum chemical energy level distributions (a susceptible substance), and the absorbed energy is subsequently dissipated, after a particular time, by reemitting light (photons) from electronically excited states. 

According to IUPAC rules, fluorescence may be defined as the spontaneous emission of light radiation from an excited entity with retention of spin multiplicity [36]. *Nota bene*: the spin multiplicity is defined as the number of possible orientations, calculated as 2S+1, of the spin angular momentum corresponding to a given total spin quantum number (S) for the same spatial electronic wavefunction. The average time these species spend in the excited state is called the fluorescence lifetime and the photon’s energy or generally a quantum of light follows from Planck’s law [39]:

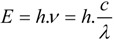
(1)
where *E* is the quantum’s energy (J), *h* is Planck's constant (J.s), *ν* the frequency (s^−1^), *λ* is the wavelength of the photon (m), and *c* is the speed of light (m.s^−1^). Because of the internal energy decay at the excited state levels (Figure 2B) and since the wavelength varies inversely with the radiative energy (Equation 1), fluorescence emission generally occurs at longer wavelengths and concomitant lower energy than the light used to excite the fluorochrome. The term “fluorescence” was first introduced by the British scientist Sir George Stokes who observed fluorescence when irradiating fluorspar (fluorite) with UV radiation and a red-shift in the resulting emitted light, which he reported in his 1852 publication “on the change of refrangibility of light” [40]. The difference between the emission and excitation maxima is called “Stokes shift”.

Phosphorescence is often phenomenologically described as being longer-lived than fluorescence, which disappears simultaneously with the end of the excitation. However, this is only partially correct, because there are short-lived phosphorescent species, such as zinc sulfide (violet), which have lifetimes comparable to fluorescent species. However, in phosphorescence, the excited species passes through an intermediate state via intersystem crossing (Figure 2B). Phosphorescence thus requires a change in spin multiplicity, from singlet to triplet state or *vice versa* (see the spin arrows in Figure 2B), whereas in fluorescence this multiplicity^1^ is retained. The subsequent relaxation from the meta-stable triplet state *ET*_1_ to the ground state *GS*_0_ is, because of the necessary spin reversal, forbidden and therefore commonly several orders of magnitude slower than fluorescence. For this reason, many phosphorescent species emit their light for prolonged periods of time; the most illustrative example are the phosphors used in dials and indices of wrist watches.

Fluorescence as a phenomenon is a complicated physical process, with numerous alternate pathways of energy conversion and/or dissipation, or environmental influencing of the final luminescent outcome. These include, non-radiative decay processes (intersystem crossing, internal conversion, predissociation, dissociation, and external conversion), quenching, energy and charge transfer, fluorescence anisotropy, intermittency, and photobleaching, to name but a few. These phenomena directly affect the emission spectrum (form and maxima), fluorescence intensity and number of photons emitted per unit time, and fluorescence lifetime. A concise introduction on fluorescence, fluorescence phenomena and artifacts, conventional single photon, confocal, two-photon and super-resolution fluorescence microscopy is provided in reference [41]. For more detailed information on fluorescence and its phenomena, the reader is referred to the “bible” of fluorescence: Lakowicz’s “*Principles of fluorescence spectroscopy*” [37]. Equally, advanced information on fluorescence microscopy can be found in Pawley’s: “*Handbook of biological confocal microscopy*” [21].

### 2.2. Advanced Fluorescence Microscopic Techniques

As stated in §2.1, fluorescence is influenced by numerous phenomena that may cause artifacts; a fact that should be taken into account when planning experiments. Nonetheless, the competent and well trained researcher will be able to handle such artifacts in order to prevent serious perturbations of the results and misinterpretation of data. However, what is most fascinating is the fact that clever researchers have always been able to turn a technological down-side to an advantage. Thus, what might be experienced by one researcher as a disadvantage and unwanted “artifact”, *e.g.*, photobleaching or intensity loss via resonance energy transfer, the same feature may be cleverly used by another to solve her/his scientific question, *e.g.*, to study diffusion of molecules via Fluorescence Recovery After Photobleaching (FRAP) or molecular interactions via Förster Resonance Energy Transfer (FRET). 

Recently, Helen Ishikawa-Ankerhold and I reviewed the basic concepts of these advanced fluorescence microscopy techniques, their utilization and value to cell biological research, and new developments in the field [41]. Basically, fluorescence microscopy-based methods to determine molecular interactions, molecule movement, whether by molecular diffusion or active transport, or a combination thereof, are based on energy or charge transfer phenomena or on methods that selectively and spatially impede fluorescence; either permanently or reversibly. The gold standard for imaging interactions between biomolecules is based on the aforementioned energy transfer between fluorescently labeled or fluorescent molecules. This photophysical process occurs when the excited state energy from a donor fluorochrome is transferred via a non-radiative mechanism to a ground state acceptor chromophore via weak long-range dipole–dipole coupling. First described mathematically by Theodor Förster in the 1940s [42,43], it requires that the donor’s emission spectrum overlaps the acceptor’s absorption spectrum and that donor and acceptor are in close proximity.

To determine the movement or transport of biomolecules, photobleaching-based or photoswitching-based methods are used. A wide variety of bleaching methods, including FRAP, Inverse FRAP (iFRAP), Fluorescence Loss in Photobleaching (FLIP), and Fluorescence Localization after Photobleaching (FLAP), have been used to determine the diffusion or active transport of biomolecules, the connectivity between different compartments in the cell or the mobility of a molecule within the whole compartment, and the mobility of molecules in small areas of an organelle, particularly the nucleus, and their exchange with the surrounding environment, and other applications. An enhancement or addition is provided by using fluorescent proteins that can be switched, either irreversibly “on” or “off” (photoactivation), from one color to another (photoswitching) or reversibly on/off, as in photochromic proteins (see Reference [41]). The advantage lies in the fact that less toxic compounds are produced (reactive oxygen species formation is always associated with photobleaching), the aforementioned proteins offer more precise localization of fluorescence, the labeling can in principle be well-controlled in a spatio-temporal manner, and fast moving sub-populations can be detected. Furthermore, by combining techniques such as FRAP and FRET, interactions between moving biomolecules can be imaged with high resolution. Such measurements would not be possible with conventional biochemical and cell biological assays.

### 2.3. Chemically induced Photoswitching of Fluorescent Probes for Super-resolution Microscopy

Optical microscopy is generally limited in its maximal resolution by aberrations caused by the various media that the light passes through, *i.e.*, diffraction. Ernst Abbe formulated the theoretical foundations for this limitation by diffraction in 1873: the smallest resolvable distance between two points cannot be smaller than half the wavelength of the imaging light [29]. The Abbe diffraction limit stood firm for more than a century, until evolving knowledge of the mechanisms of fluorescence allowed researchers to “cheat” the diffraction limit by choosing circumstances in which this limit no longer was valid. For instance, stimulated emission depletion (STED [44]) is a technique in which an initial “broad” focal spot is shrunk in its diameter (below the diffraction limit) by depleting the outer excited state fluorochromes through stimulated emission with a doughnut-shaped STED beam that is red- and △t time-shifted (modulation of transitions between two states). This represents one category of super-resolution approaches. Alternatively, a number of techniques are based on the temporal confinement of fluorescence and the precise spatial localization of individual fluorochromes by repeated photoswitching of a limited number of fluorochromes in the total pool from which a super-resolution image can be reconstructed. These include structured illumination approaches (SIM [45]), Photo-Activation Localization Microscopy (PALM [46]), STochastical Optical Reconstruction Microscopy (STORM [47,48]) and others. 

Techniques such as PALM and STORM rely on the use of fluorescent probes that can be switched reversibly between a fluorescent “on” and dark “off” state or at least can be photoactivated. In STORM, originally a cyanine switch was used; a pair of orange and red-emitting carbocyanine dyes, Cy3 and Cy5, in which Cy5 can be reversibly switched between fluorescent and dark states provided that a second activator dye, Cy3, is in close proximity. A major disadvantage of STORM is that most organic probes used in STORM preclude imaging in living cells, because they require the removal of molecular oxygen or need a reducing environment, which puts the cell in a state of extreme stress. Direct STORM (*d*STORM), a variation of the original technique, does not require the use of paired photoswitches, but uses conventional stand-alone carbocyanine dyes (*e.g.*, Cy5, Alexa Fluor 647, and several dyes from the ATTO series). A major advantage is that these carbocyanine dyes can be used in living cells in combination with site-specific and targeted labeling of the biomolecule of interest. PALM on the other hand uses fluorescent proteins and thus has the advantage of genetically co-expressing the label with the protein of interest, at the required location (intracellularly and on the protein of interest) without the need to disrupt membranes and with negligible perturbation of cellular homeostasis. An extrapolation of the probes used in PALM or combination with the *d*STORM approach might significantly improve super resolution live cell imaging in the presence of molecular oxygen.

For this reason, Ulrike Endesfelder from Mike Heilemann’s group used the basic knowledge of the mechanism of photoswitching in organic fluorochromes (under *d*STORM reducing conditions) to improve super-resolution imaging and allow its application in living cells [49]. They investigated if fluorescent proteins, *i.e*., PAmCherry1 (photoactivatable), mEos2, Dendra2 and psCFP2 (all photoconvertible), and bsDronpa (photoswitchable), might be used for live cell imaging under *d*STORM conditions *per se*, the required and optimal environmental conditions, and whether these proteins can be used in combination with organic dyes for dual-color super-resolution imaging. 

### 2.4. Twisted Intramolecular Charge Transfer and Excimer Emission in 2,7-bis(4-Diethylaminophenyl)-fluorenone

Energy transfer phenomena, such as FRET, have extensively been applied to study molecular interactions, conformational changes in molecules, as probes in reporter assays, or more recently in organic semiconductors, such as OLEDs (organic light-emitting diode). Next to FRET, in which energy is transferred between susceptible molecules, charge or electron transfer can also deplete the excited state, thereby changing the fluorochrome’s fluorescent properties. Dexter electron transfer (DET), for instance, is a process in which two molecules (intermolecular) or two parts of the same molecule (intramolecular) bilaterally exchange their electrons [50]. Unlike FRET, DET takes place at much shorter distances. Charge transfer processes include excimer and exciplex formation [41], which are short-lived homodimers (excimer) or heterodimers (exciplex) of which at least one molecule is in the excited state. Such complexes occur via electrostatic attraction because of partial charge transfer between the individual entities and show red-shifted emission compared with the monomer’s emission.

**Figure 3:**
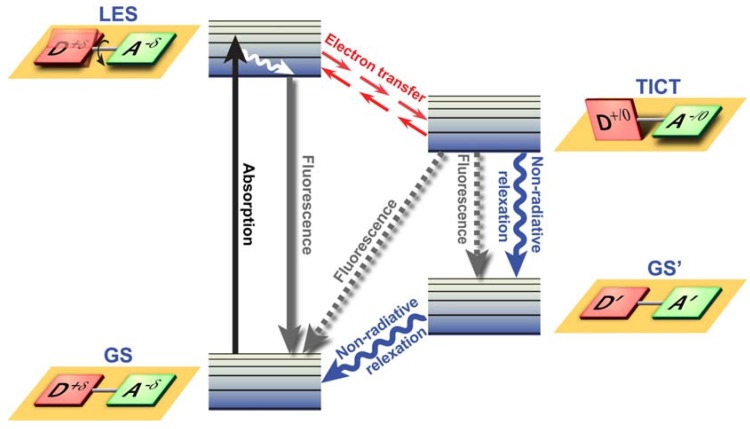
Twisted Intramolecular Charge Transfer (TICT) dynamics. Upon excitation from the ground state (GS), the locally excited state (LES) equilibrates rapidly with the TICT state after fast electron transfer. The TICT state is energetically lower and relaxation from the TICT state occurs either radiatively or non-radiatively to a thermally hot ground state (GS’) which after dissipation of heat becomes the GS. Alternatively, excess energy is dissipated via direct radiative relaxation to the GS. The exact pathway for energy dissipation depends strongly on the polarity of the environment.

Twisted Intramolecular Charge Transfer (TICT) is a relatively common phenomenon in molecules that consist of an electron donor and acceptor pair linked by a single bond [51]. In polar environments, such fluorochromes undergo fast intramolecular electron transfer from the donor to the acceptor part. This electron transfer is subsequently followed by intramolecular twisting of donor and acceptor about the single bond (Figure 3) and produces a relaxed perpendicular structure and emits dual fluorescence, *i.e*., from a high energy band through relaxation of the locally excited state and from a lower energy band due to emission from the TICT state. However, since there are a number of relaxation pathways, it would be highly desirable to control the emissive relaxation from the TICT state, since TICT fluorescence holds great promise in applications such as OLEDs, chemosensors, and dye-sensitized photovoltaic applications.

To further pursue the objective of obtaining switchable molecules, Konishi’s group recently developed a donor-acceptor-donor dye consisting of a 2,7-disubstituted fluorenone with diethyl-aminophenyl moieties as strong electron donating groups [52]. This novel dye can easily be switched between TICT and excimer emission via the polarity of the surrounding solvent without any ground state changes. The authors hypothesized that when excimer emission was observed, either the TICT state was not formed, or once the TICT was formed, it was converted into an excimer by a Coulombic force acting between opposite charges, such as in the Harpooning effect [53]. The development of this new dye or other dyes like it might in future applications lead to the construction of sensors that provide information on solvent polarity in real-time or as part of quick-tests.

### 2.5. Fluorescence Quenching to Study Binding of Flavonoids to Bovine Serum Albumin

In a recent publication, Lui *et al.* [54] showed how fluorescence quenching can effectively be used in biological research to determine the binding mechanisms of phytochemicals to serum proteins. In this study, the authors investigated the interaction between five flavonoids, *i.e*., the polyphenols formononetin-7-*O*-β-D-glucoside, calycosin-7-*O*-β-D-glucoside, calycosin, rutin, and quercetin, and bovine serum albumin (BSA). By utilizing BSA’s intrinsic ability to fluoresce (autofluorescence), they were able to show that formation of a flavonoid-BSA complex led to quenching of BSA’s autofluorescence. Fluorescence quenching occurs, because the molecular species (quencher) that is in close proximity depletes the excited state of the fluorochrome by non-radiative mechanisms (Figure 2B), thereby reducing the quantum yield and/or the lifetime.

To provide a quantitative measure for the binding affinity, fluorescence quenching constants were determined using the Stern-Volmer and Lineweaver-Burk equations. Based on these fluorescence quenching constants, the compounds ranked in the following order: quercetin > rutin > calycosin > calycosin-7-*O*-β-D-glucoside ≈ formononetin-7-*O*-β-D-glucoside. Thermodynamic evaluations demonstrated that hydrophobic interactions played a major role in the flavonoid-BSA interaction. Mechanistical studies suggested that flavonoid-BSA quenching occurred through static quenching – direct interaction of the fluorochrome and the quenching molecules, for instance by forming a non-fluorescent ground state complex. 

To further substantiate their findings, the authors performed FRET measurements and determined the distance *r* between BSA (donor) and the aforementioned flavonoids (acceptor). The values for *r* were 4.12 for formononetin-7-*O*-β-D-glucoside, 3.85 for calycosin-7-*O*-β-D-glucoside, 3.01 for calycosin, 5.72 for rutin, and 4.75 nm for quercetin and therefore demonstrated a close interaction between the flavonoids and BSA. A comprehensible review on Förster’s theory of non-radiative energy transfer, including references to more specialized and comprehensive overviews, was recently provided by Ishikawa-Ankerhold *et al.* [41].

### 2.6. Molecular Morphology of Pituitary Cells: Immunohistochemistry to Fluorescence Imaging

Electron microscopy-based (EM) *in situ* hybridization (ISH) is an essential technique for studying a biomolecule’s intracellular distribution and its role in both normal and abnormal cellular behavior. Combination of ISH and immunohistochemistry (IHC) with EM (EM-ISH & IHC) provides sufficient ultrastructural resolution to evaluate the intracellular localization of even small biomolecules, such as mRNA. With the development of nanoparticles (§3.7), especially semi-conductor quantum dots (Qdots), it is now possible to obtain sufficient optical signal from individual biomolecules in confocal laser scanning microscopy (CLSM), albeit with less resolution than EM. 

Matsuno and co-workers scrutinize the developments from conventional immunohistochemistry to fluorescence imaging, with a particular focus on the intracellular localization of mRNA and the exact site of pituitary hormone synthesis on the rough endoplasmic reticulum in pituitary cells. In their paper, not only ISH, IHC, CLSM and EM techniques are discussed, but they show that both EM-ISH&IHC and ISH& IHC using Qdots and CLSM are useful for understanding the relationships between protein and mRNA simultaneously in two or three dimensions. Furthermore, they developed an experimental pituitary cell line (GH3), in which the growth hormone (GH) is linked to enhanced yellow fluorescent protein (EYFP). The GH3 cell line secretes the GH‒EYFP fusion protein upon stimulation by Ca^2+^ (influx or release from storage) and allows the real-time visualization of the intracellular transport and secretion of GH. This approach from conventional immunohistochemistry to fluorescence imaging allows researchers to consecutively visualize the processes of transcription, translation, transport and secretion of the anterior pituitary hormone.

## 3. Fluorochromes

### 3.1. Pyrene: A Probe for Protein Conformation studies

**Figure 4:**
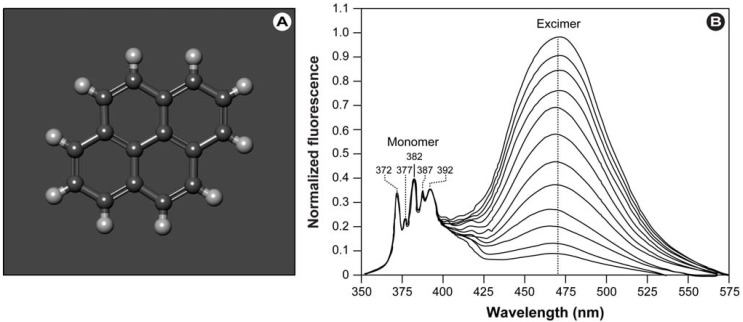
Pyrene’s excimer formation in lipid vesicles. (**A**) Molecular structure of pyrene (Benzo[d,e,f]phenanthrene; C_16_H_10_). (**B**) Emission spectra of pyrene in egg-PC. Spectra are normalized to the 372 nm peak of the monomer and excimer formation can increasingly be observed at ~460 nm with increasing pyrene concentration.

Pyrene (Figure 4A) is one of the most widely spread and oldest fluorochromes used in cell biology/biophysics. Pyrene-based probes have commonly been used to study membrane fusion, lipid domain formation, lipid transport mechanisms, lipid-protein interactions with FRET, in photodynamic therapy, nucleic acid dynamics, and protein conformation and conformational changes, to name but a few. Pyrene can easily be incorporated into phospholipids, either by substitution at the *sn*-2 position or biosynthetically by growing cells in the presence of pyrene fatty acids [55,56]. Furthermore, proteins can be site-specifically labeled on lysines succinimidyl ester, isothiocyanate and sulfonyl chloride group reactivity or cysteines with maleimide and iodoacetamide group reactivity.

It has long been known that pyrene’s fluorescent and spectral properties are highly sensitive to changes in the probe’s microenvironment. Besides utilizing the characteristics and changes in the monomer emission bands (350–400 nm; Figure 4B) for polarity measurements of the microenvironment, the formation of a broad excimer–excited state dimer of two interacting pyrene molecules–emission peak at ~460 nm can be utilized to study protein conformation, conformational changes, protein folding and unfolding, protein-protein, protein-lipid and protein-membrane interactions.

In a recent overview, Bains *et al*. discuss the intrinsic fluorescence properties of pyrene, the mechanism of excimer formation and how to extract information from these to study protein conformation and conformational changes [57]. With this review, the authors provide insightful information for the interested researcher.

### 3.2. Li^+^ Selective Podand-Type Fluoroionophores

The sensing of ions is important in a myriad of scientific and technological disciplines, including biology, (bio)medicine, and environmental chemistry. Common strategies include molecules that have ion recognition units, such as crown ethers or other complexing structures, which upon ion binding induce changes in the absorption and/or fluorescence behavior of the attached fluorochrome. Recently, we designed a luminescent lanthanide complex-based anion sensor with electron-donating methoxy groups for concomitant monitoring of multiple anions, including fluoride, acetate and dihydrogen phosphate [58]. Metal ion sensing is equally important, especially since they play key roles in biological and envirmonmetal systems. 

Nishimura *et al*. designed podand-type fluoroionophores in which the ion recognition unit is coupled to pyrenyl groups connected by appropriate linkers [59]: 2,2′-bis(1-pyrenylacetyloxy)-diphenyl sulfide (**3**), sulfoxide (**4**), and sulfone (**5**). These were partially sensitive to alkali metal ions (Li^+^, Na^+^, K^+^, Rb^+^, Cs^+^) and binding induced a characteristic change in their emission spectra. Most importantly, compound (**4**), which contains a sulfinyl group as the non-cyclic binding site, effectively reacted to Li^+^ ion binding and would constitute a suitable Li^+^ fluorescence sensor.

### 3.3. Molecular Dynamics Simulations of Fluorescent Membrane Probes

Probing biomembrane dynamics, structure, and membrane-based cellular physiology is commonly performed with fluorescent probes and by closely observing changes in the spectrum, fluorescence lifetime, quantum yield, and by measuring double labeled constituents to determine FRET. A variety of fluorescent probes, such as the aforementioned pyrene (§3.1), are generally used to study the biophysical behavior of biomembranes, because of their high sensitivity, versatility, and sub-nanosecond time resolution. Such probes are either inserted into the lipid bilayer or covalently attached to lipids. However, depending on the particular probe used, local or wide-spread perturbations of the biomembrane, *i.e*., disruption of the bilayer, dynamics of bilayer constituents and bilayer thermotropics, may occur and thus experiments with probe-based methods might be compromised. Therefore, it is essential to understand such perturbations and to develop probes that will minimally interfere with normal biomembrane properties and homeostasis.

Over the past decade, molecular dynamics simulations (MDS) have been developed to analyze the location and dynamics of the inserted probe and its effect on the bilayer. Until recently, these MDS were based on simple atomistic simulations of non-polar probes in fluid disordered bilayers. However, the field has not been stagnant in its development, but rather moved towards improved and more intricate MDS methodologies that allow simulation of increasingly complex fluorochromes and extension of MDS in ordered bilayers, particularly containing cholesterol (an important regulator of membrane fluidity in mammalian cells). Consequently, Loura and Ramalho [60] review these developments to provide easy access to new developments for a broad life science audience. They show that a dramatic increase and diversification of MDS has taken place, with reported studies in all common lamellar lipid phases (liquid disordered, liquid ordered and gel phases). Simple apolar probes such as DPH and the aforementioned pyrene (see §3.1) have been the focus of study, but recent emphasis has shifted to complex amphiphilic probes, *e.g.*, NBD, BODIPY, rhodamine or cyanine dyes. 

### 3.4. Fluorescent Lipids in Fusogenic Liposomes for Cell Membrane Labeling and Visualization

Biomembranes represent important structures in cells. Not only do they ensure compartmentalization so that the multitude of biological and chemical processes are separated, provide a barrier against the harsh extracellular environment, only allow particular molecules to enter and leave the cell, are storage places for signaling molecules and energy, selectively transport both signals and biomolecules within the cell, but the plasma membrane is also the largest organelle in the cell. Disruption of these processes might cause disease and not surprisingly, membranes and their dynamics have been to focus of intense research. To label biomembranes, fluorescently labeled lipids are commonly used. These, however, suffer from a number of drawbacks, depending on the label and concentration used: (i) large fluorochromic groups might perturb the biomembrane or might not represent the endogenous motility; (ii) labeling of living cells is difficult, except when the membrane label is incorporated biosynthetically during cell culture [61,62]; (iii) labeling procedures might induce cellular stress and therefore perturb the experimental results, and iv) generally, the labeling efficiency is low. The latter restriction was overcome with the introduction of fusogenic liposomes. These induce membrane fusion with the plasma membrane and contain neutral and positively charged lipids.

To surmount the majority of the aforementioned drawbacks, Kleusch *et al.* developed a method in which novel combinations of fluorescent lipid derivatives in fusogenic liposome carriers are utilized [63]. The authors specifically used a combination of a biologically irrelevant fluorescent component that triggers membrane fusion at a concentration of 2−5 mol%, *e.g*., DiR, and a second, biologically active fluorescent component, *e.g*., sphingomyelin-BODIPY-FL. DiR (1,1'-dioctadecyl-3,3,3',3'-tetramethylindo-tricarbocyanine iodide), is a near IR fluorescent, lipophilic carbocyanine that is weakly fluorescent in water but highly fluorescent and photostable when incorporated into membranes [35]. As the authors express it, the primary advantage of a combined fusogenic delivery system is the controlled delivery of fluorescent molecules in a broad concentration range. Furthermore, this research shows that a significantly improved fluorescent signal can be obtained, with excellent signal to noise ratios.

### 3.5. Oligothiophenes as Fluorescent Markers for Biological Applications

Oligothiophenes are a class of organic molecules that are conveniently and flexibly produced by coupling of repeating thiopene monomers (C_4_H_4_S; five rings with a central S), most common via oxidative homocoupling or metal-catalyzed C-C-coupling, *e.g*., Kumada, Suzuki, or Negishi-based. Because virtually any form can be built, large conjugated π-systems can be produced with controllable electronic properties over a wide range. Besides this potential for structural variation, oligothiophenes have unique electronic, optical, and redox properties, show unique self-assembling properties on surfaces or in bulk, and the high polarizability of sulfur atoms in thiophene rings leads to stabilization of the conjugated chain and to excellent charge transport properties [64]. Oligomers of thiophene are widely used in organic electronics, such as OLEDs, because of their semiconductor properties. In biological applications, especially for labeling DNA, oligothiophenes have gained much interest over the past decade, particularly because their fluorescent properties can be modulated by varying the number of thiophene rings and the nature of the side-chains. 

Capobianco *et al*., extensively discuss the use of oligothiophenes as fluorescent probes in biological applications [65]. Their review addresses the derivatization of oligothiophenes with active groups, such as phosphoramidite, *N*-hydroxysuccinimidyl and 4-sulfotetrafluorophenyl esters, isothiocyanate and azide, in order to covalently label the biomolecule of interest, especially DNA. Furthermore, the authors describe how functionalized oligothiophene probes can be used in hybridization studies and bio-imaging. 

### 3.6. Phthalocyanines in Biomedical Optics

**Figure 5:**
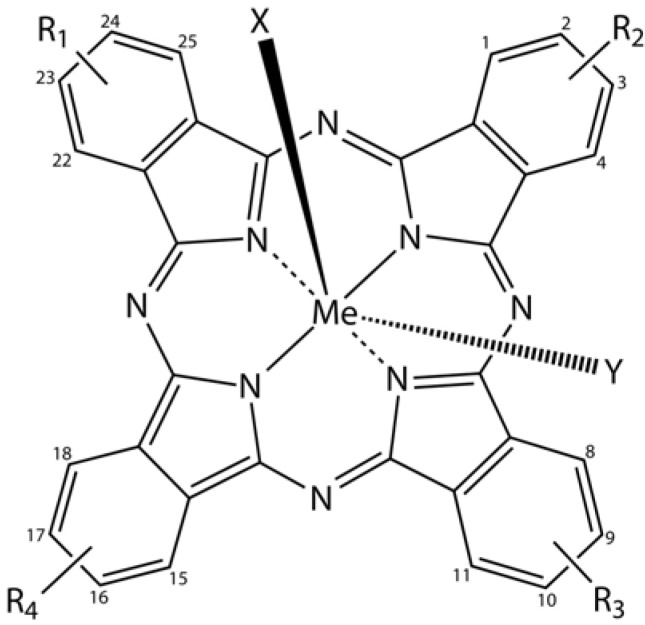
General chemical structure of metal-phthalocyanine dyes. Me denotes the metal ion; R_1-4_ are peripheral functional groups and X and Y are optional functional groups above and below the molecule’s plane (axial).

Phthalocyanine derivatives (PcDer) have extensively been used in various dye-based applications, since they show intense green to blue colors, depending on the functional group and the complexed metal ion. Approximately one quarter of all synthetically produced organic pigments are PcDers [66] and thus this class of dyes is widely applied in industrial applications, such as paints, printing ink, leather, textile and paper dyeing. In biomedical science, a multitude of PcDers have been developed and are under increasing investigation as photosensitizers (PS), amongst others for photodynamic therapy, and as imaging agents in bio-imaging. PcDers are porphyrin-like PS, consisting of tetrapyrrolic nitrogen-linked aromatic macrocycles, which have high extinction coefficients around 670 and 750 nm. Their properties, such as fine-tuning of NIR absorbance, pharmacokinetics, biodistribution, solubility, and stability can be directly controlled via the axial and peripheral substituents (Figure 5).

In a recent review, Norbert Lange and co-workers [67] present a comprehensive overview of the use of PcDers in photodynamic therapy, as imaging agents, their pharmacological and therapeutic significance, and critically address some of the shortcomings and how to overcome these.

### 3.7. Fluorescent Nanoprobes for in vivo Imaging

The convergence of nanotechnology–the construction, manipulation, and utilization of materials at nanoscale dimensions [68]–and biotechnology into nanobiotechnology has produced a multitude of (semi)synthetic nanoparticles with entirely new possibilities for biological investigations and potentially performing medical interventions at the (sub)cellular level, *i.e*., nanomedicine. Nanoparticles offer significant advantages over more conventional strategies in that they display enhanced sensitivity, shorter turn-around-times, allow multiplex analysis for *in vitro* diagnostics and imaging with excellent signal to noise ratios, and potentially permit combination of imaging and targeted therapy (multimodal probes) [34]. One of the prime reasons why nanobiotechnology holds so much promise is that nanoparticles are in the same size-range as biomolecules. Most importantly, the physical properties of materials are distinctly different at the nano-scale compared with the same material in bulk form, and are size- and shape dependent. In this fashion, the fluorescent properties of nanoparticles can be directly controlled by controlling their size and shape.

Juliette Mérian from Isabelle Texier’s group provides an interesting and comprehensive overview of current developments in nanoparticle-based probe design and their application in *in vivo* imaging [69]. The authors closely evaluate the steps necessary for translation of the current generation of probes under preclinical evaluation to routine application in a clinical setting. It is expected that in the coming decades, nanoparticles may indeed be routinely used as imaging agents in diagnostics and surgical guidance, or as controlled release vehicles of surface-bound or internal bioactive payloads and as such provide a site-specific, less stressful and patient-friendly medicine with fewer side-effects.

### 3.8. Fluorescence-Based Multiplex Protein Detection Using Optically Encoded Microbeads

The concomitant detection of multiple signals for multiple biological parameters has long been at the top of the wish list of bioscience and biomedical researchers for development of multiplex assays and high-throughput screening (HTS), as well as physicians for utilization in fast and easy diagnostics. Optical methods, including fluorescence- and plasmonic phenomena-based detection, have the potential to achieve this goal, provided that the individual signals are well separated and minimal bleed-through in the various channels occurs. Technologies that use reduced sample volumes, allow the detection of multiple signals, and that use fast detection of these signals are highly suitable for the aforementioned purposes, especially HTS. 

In recent years, particularly the use of bar-coded micro-sized beads (microbeads) in bead-based suspension or liquid arrays has gained much attention for the multiplex detection of biomolecules. With their large surface area, more capture biomolecules can be immobilized on the bead’s surface compared with conventional arrays, detection is fast and the sensitivity of detection is at least equal to established methods, target molecules can be collected by using flow cytometry, the beads can be used in combination with microfluidic devices, and large-scale fabrication, easy customization and storage round up some of the advantages of this technology. In this special edition, Bong-Hyun Jun *et al*. review recent developments of analytical protein screening methods on microbead-based platforms, such as barcoded microbeads, and molecular beacon-, and surface-enhanced Raman scattering-based techniques [70]. The authors conclude that this technology has come a long way, but still is far from mature. Issues that remain to be addressed include: development of a larger number of optical codes, increased speed in the readout, safety issues, cost effectiveness, increased sensitivity, and a requirement for more ergonomic equipment for use in bioapplications and a clinical setting.

### 3.9. Fluorescence Spectroscopic Properties of Silyl-Substituted Naphthalene Derivatives

Silicon (Si) is the second most abundant element in the Earth's crust and shares group 14 of the periodic table with carbon. This tetravalent metalloid element can behave similarly to carbon and analogously may form complex molecules, *e.g*., silanes, silenes, organosilicon *etc.*, albeit that Si is less reactive than carbon. Organosilicon compounds are organic molecules that contain carbon-silicon bonds, with organically bound silicon being tetravalent and tetrahedral, are distinctly environmentally friendly and have extraordinary photochemical and luminescent properties, including photoinduced electron transfer reactions and intramolecular charge transfer complex formation in aromatic disilanes. Interestingly, substitution of organic fluorescent dyes, such as anthracenes, naphthacenes, pentacenes, and pyrenes, with silicon-bearing groups, particularly silyl, silylethynyl, but also other members of the period group, *e.g*., germyl and stannyl, induce enhancement of the fluorescence intensity.

Hajime Maeda and co-workers studied the fundamental absorption and fluorescence properties of monosilyl-group substituted naphthalene derivatives: 1-silyl-, 1,4-disilyl-, 1-silylethynyl- and 1,4-disilylethynyl-naphthalenes [71]. Their research showed that 1-silyl- and 1,4-disilylnaphthalenes show absorption maxima at longer wavelengths with larger *ε* values than those of naphthalene. Furthermore, bathochromic effects and incremental increases in *ε* were observed for electron-donating, electron-withdrawing and silylethynyl group substituted naphthalenes, and fluorescence quantum efficiencies increase, whilst lifetimes decrease when the silyl substituents are on the naphthalene ring system.

### 3.10. Fluorescent Probes for Detecting the Phagocytic Phase of Apoptosis

Apoptosis or programmed cell death is a regulated and orderly form of elimination of cells that have gone awry, have been invaded by pathogens, or were damaged by exogenous causes [72]. It is distinctly different from necrosis in that no loss of plasma membrane integrity occurs. The demolition process starts with a series of perturbations of the cellular architecture that set in motion the process of cell death, condensation and fragmentation of the nucleus, globularization, membrane blebbing, detachment from the surrounding cells, and preparation for recognition and removal by phagocytes. Furthermore, unwanted immune responses are prevented. The apoptotic corpses are subsequently cooperatively removed by phagocytic cells and this phase of apoptosis ensures efficient degradation of DNA, which in turn inhibits self-immunization, inflammation, and the release of viral or tumor DNA [72,73]. During the phagocytic phase of apoptosis, DNA is degraded by a single nuclease DNase II. 

With the current technology, optical microcopy-based assessment and detection of phagocytizing cells, and accurate discrimination of adherent versus internalized apoptotic cells is challenging and labor-intense. Therefore, the development of fluorescent probes that are capable of detecting this phase of apoptosis is highly desirable in order to allow researchers to better understand the basic processes involved. A major step towards achieving this goal was recently made by Candace Minchew and Vladimir Didenko. These authors synthesized fluorescent probes that are the covalently-bound enzyme-DNA intermediates produced in a topoisomerase reaction with specific “starting” oligonucleotides; composition: vaccinia topoisomerase-I−hairpin-shaped oligonucleotide–probe (fluorescein isothiocyanate) [74]. The probe selectively detects blunt-ended 5’OH DNA breaks, which are specific markers of DNase II cleavage activity. In sections and fixed cells, this methodology allows the imaging of digestion processes that occur in cellular organelles, which are responsible for the actual execution of phagocytic degradation of apoptotic cell corpses. The authors applied the probes to visualize and study the phagocytic reaction in tissue sections of normal thymus and in several human lymphomas.

### 3.11. Fluorescent Hyaluronan Analogs for Hyaluronan Studies

Hyaluronan or hyaluronic acid (HA) is an anionic, nonsulfated, linear, high molecular weight polyglycosaminoglycan, consisting of repeating units of the disaccharide D-glucuronic acid-β(1→3)-*N*-acetyl-D-glucosamine-β(1→4). *In vivo*, HA can be found in a wide variety of tissues with varying molecular weights, ranging from 5,000 to 20,000,000 Da, *e.g*., in human synovial fluid 3−4 million Da, in human umbilical cord 3,140,000 Da [75]. Hyaluronan is ubiquitously present in the extracellular matrix of all vertebrates and the capsule of group A Streptococci. About 50% of HA is found in the skin and 25% in the skeleton and its supporting structures, such as ligaments and joints (here it acts both as a lubricant and is responsible for the compressive properties of articular cartilage).

**Figure 6:**
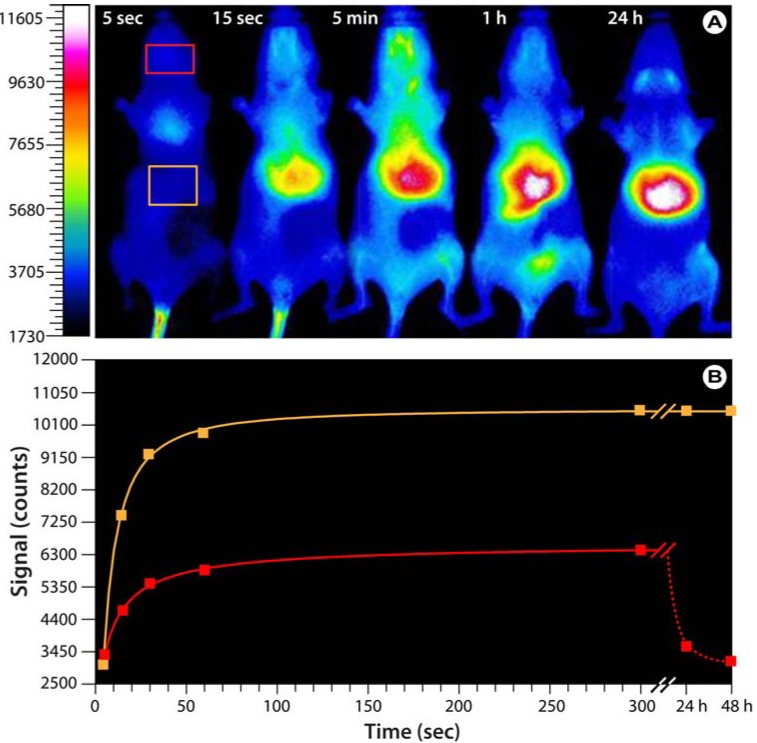
Bio-imaging with hyaluronan analogs modified with the near-infrared IR-783 dye.(**A**) Optical images of SKH mice injected with fluorescent HA-NIRdye (1% dye load). Based on Reference [76]. Note that the probe predominantly accumulates in the upper abdominal and thoracic organs, *i.e.*, liver, heart, lungs, kidneys, stomach, and lymph nodes. After 24−48 h, the probe is exclusively found in the liver. (**B**) Progression of the fluorescence intensity in the boxes indicated in A.

HA is synthesized on the inner face of the plasma membrane instead of the Golgi, and directly extruded into the extracellular matrix. Since HA is involved in a myriad of normal and abnormal biological processes, including cell proliferation and migration, wound repair, the aforementioned functions in cartilage, maintenance of the hydration and osmotic balance of tissues because of its high water binding capacity, and plays a role in certain cancers, *e.g*., mesothelioma, Wilms’ tumor, prostate and breast cancer, and in bladder cancer, HA was found to be associated with tumor angiogenesis and metastasis [77]. Furthermore, HA is widely used in cosmetics, especially skin-care products, and in cosmetic surgery as dermal filler. Therefore, studying the role of HA in both physiological and pathophysiological function is a highly relevant and attractive topic.

To enable imaging of various processes and applications involving HA, suitable HA-based probes must be developed. For this purpose, Wei Wang from Shi Ke’s group developed fluorescent HA analogs based on the near-infrared heptamethine cyanine dye IR-783 for cellular and small animal imaging applications [76]. The researchers developed two different forms of the HA analogs; one for normal imaging purposes and a modified version as a biosensitive contrast agent by labeling HA with varying molar percentages of IR-783. At low labeling ratios, the uptake and transport of hyaluronan can be directly imaged while at high labeling ratios, the fluorescent signal is quenched and fluorescence emission only occurs after HA degradation within the cell. Preliminary investigations in hairless SKH mice not only show a rapid distribution after tail vein injection and subsequent accumulation in various glandular systems and upper abdominal and thoracic organs (Figure 6), but also the feasibility of using these HA analogs in whole animal imaging.

## 4. Concluding Remarks

Luminescent technology has undeniably been a bright light in human development and science. Especially the past few decades have seen major advancements with the discovery of fluorescent proteins, novel small animal imaging methods, super-resolution microscopy, lasers and LEDS, to name but a few. Interesting enough, there seems to be no limit to the innovation in luminescent technologies, with holographic imaging and display, white light super-resolution microscopy with nano-lenses, and a myriad of OLED applications on the horizon. The next decades will certainly be extremely exciting for those of us working at the interface of nanoscience, chemistry, medicine, and biology. The papers presented in this special edition show the intensity of the research efforts in this field of science and biomedical/biological science researchers are certainly going to benefit from these innovations. However, the results here also highlight some of the disadvantages that still remain. It is therefore essential for researchers to have a profound knowledge of the basic principles involved in photoluminescence. The myriad of high quality reviews that appear regularly will certainly aid to achieve this goal. The future is bright!
